# Frailty predicts increased resource use and postoperative care requirements after revision hip surgery

**DOI:** 10.1186/cc14624

**Published:** 2015-03-16

**Authors:** A Panickar, N Singatullina, J Stubbs, C Johnson, R Porter, D Bryden

**Affiliations:** 1 Sheffield Teaching Hospitals, Sheffield, UK; 2 Leicester Hospitals, Leicester, UK

## Introduction

There is increasing demand for revision hip surgery in older patients with poor frailty. Our previously submitted work demonstrated that frailty predicts the need for medical review [1]. We reviewed patients for a further 16 months to see whether frailty impacts on care [2]. This is the largest reported study reviewing frailty and the need for organ supports and outcomes in complex orthopaedic surgery.

## Methods

A retrospective note review of all patients from January 2012 to April 2014 undergoing revision hip surgery. Data collected included frailty, comorbidities, operative blood loss, anaesthetic technique and level of organ supports and patient location at 30 days.

## Results

**Figure 1:**
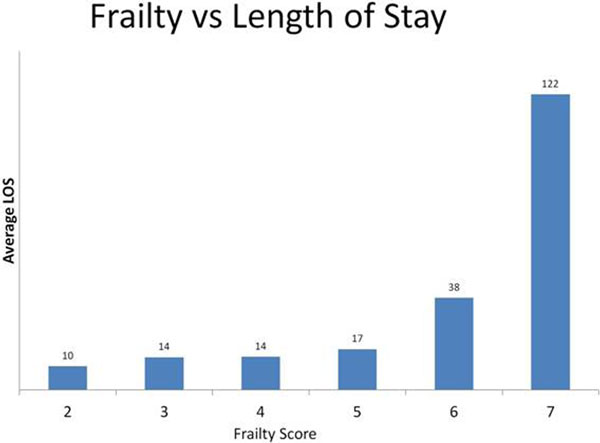
**Frailty versus length of stay**.

A total of 389 patients with a mean age of 68.7 years were identified. Frail patients were significantly more likely to need vasopressors postoperatively (*P *= 0.012). Each increase in frailty score was associated with 0.16 increase in length of stay on the HDU (*P *= 0.025). Analysis of patient location at 30 days shows that frail patients stay in hospital longer (*P *= 0.00). Frail patients also bleed more intraoperatively (*P = *0.00 with a coefficient value of 239; that is, for every point increase in frailty, average blood loss increases by 239 ml). For each increase by unit of blood transfused, the length of stay increased by 5.3 days (*P *= 0.000). The use of epidural is not associated with increased need for postoperative vasopressors (*P *= 0.598). See Figure 1.

## Conclusion

Frailty is associated with increased intraoperative resource use and postoperative care requirements independent of choice of anaesthetic technique. This type of surgery should be subject to health economic analysis as demand amongst the frailer surgical population increases.
